# Regulation of Pickling Efficiency and Physicochemical Properties of Reduced-Sodium Chicken Breast Using Shiitake Mushroom Extract and Ultrasound-Assisted Marination

**DOI:** 10.3390/foods15071207

**Published:** 2026-04-02

**Authors:** Shuqiang Zhang, Yungang Cao, Min Li, Bin Yu, Haiteng Tao, Zhengzong Wu, Xuemin Kang, Guimei Liu, Lu Lu, Feixue Zou, Haibo Zhao, Bo Cui

**Affiliations:** 1 Shandong Key Laboratory of Healthy Food Resources Exploration and Creation, School of Food Science and Engineering, Qilu University of Technology, Shandong Academy of Sciences, Jinan 250353, China; 2 State Key Laboratory of Green Papermaking and Resource Recycling, Qilu University of Technology, Shandong Academy of Sciences, Jinan 250353, China; 3 School of Food Science and Engineering, Shaanxi University of Science & Technology, Xi’an 710021, China; 4 Shandong Delisi Food Co., Ltd., Weifang 262216, China

**Keywords:** chicken breast, reduced-sodium marination, shiitake mushroom extract, ultrasound, texture, water retention

## Abstract

Reducing sodium in poultry products often compromises texture and water-holding capacity. This study investigated the interactive effects of shiitake (*Lentinula edodes*) mushroom extract (SME) and ultrasound (US) on mitigating these defects in reduced-sodium chicken breast. A standard 2.0% NaCl brine served as the control, while reduced-sodium formulations contained 1.4% NaCl supplemented with 0.2% or 0.6% KCl. SME (0.4–1.2%) and probe US (20 kHz, 300 W, 15 min) were applied. Independently, SME increased brine uptake (15% at 1.2% SME), while US accelerated chloride diffusion (~30%) via microstructural disruption. The synergistic treatment combining 0.8% SME with US was identified as the statistical optimum. Simple effect analysis confirmed this combination significantly reduced cooking loss (*p* < 0.01) and restored comprehensive textural attributes to levels comparable with the 2.0% NaCl control. As confirmed by LF-NMR and microstructural analysis (H&E staining), these macroscopic improvements were highly correlated with optimized immobilized water (*P*_21_) proportions and myofibrillar protein-matrix stabilization. Ultimately, this strategy provides a functional and microstructural basis for developing sodium-reduced poultry without compromising critical quality attributes.

## 1. Introduction

The causal relationship between excessive sodium intake and the prevalence of hypertension and cardiovascular disease has intensified the public health mandate for reduced-salt diets, placing processed meat products under sustained scrutiny [1,2,3]. Among these products, poultry—particularly chicken-based products such as nuggets—remains a major global dietary staple because of its cost-effectiveness, broad consumer acceptance, and high-quality protein content [4]. In poultry processing, NaCl is not merely a flavoring agent; it is also a critical functional ingredient that improves water-holding capacity, protein extraction, texture, and overall eating quality. Although NaCl levels in commercial poultry products are often reported in the range of 1.0–1.6 g/100 g, formulations may increase salt addition (e.g., ~2.5 g/100 g) to secure processing stability and sensory quality, making poultry products an important target for sodium reduction strategies [5,6].

Historically, the partial replacement of NaCl with potassium chloride (KCl) has been the most direct strategy for reducing sodium reduction. However, practical implementation is frequently limited by KCl-associated metallic and bitter off-notes, especially at higher substitution levels [7]. To mitigate these sensory constraints, researchers have explored a range of approaches, including modification of salt crystal size/structure and incorporation of plant-based ingredients such as macroalgae; nevertheless, identifying a “clean-label” strategy that simultaneously maintains saltiness perception, texture, and consumer acceptability remains challenging [8,9,10].

A promising direction is the strategic use of natural umami enhancers, which can synergistically increase perceived saltiness and potentially compensate for reduced NaCl without introducing artificial additives [11]. This concept aligns well with increasing consumer demand for additive-free and minimally processed foods [12]. Shiitake mushrooms (*Lentinula edodes*) are widely consumed and valued for their strong umami profile, and they contain multiple flavor-active components that may enhance savory intensity and overall palatability [13]. Mushroom-derived extracts have been used to improve flavor and sensory quality in products including beef soup [14], beef ham [15], chicken sausage [16], and Frankfurt sausage [17]. Importantly, the application of mushroom extracts extends beyond mere sensory enhancement to significant physicochemical and structural functionalities. This dual-purpose characteristic is particularly relevant because reducing sodium levels in chicken breast processing typically results in compromised myofibrillar protein solubility and a fragmented gel matrix, leading to undesirable texture and high cooking loss [18]. To mitigate these detrimental effects, the incorporation of Shiitake mushroom extract (SME) offers a multi-functional solution. Beyond its flavor-enhancing effects, SME contains various bio-macromolecules, including polysaccharides (such as β-glucans) and bioactive peptides, which can significantly influence the physicochemical stability of meat systems. From a structural perspective, these macromolecules possess numerous hydrophilic groups that can enhance water binding through hydrogen bonding. Moreover, they may interact with myofibrillar proteins via electrostatic or hydrophobic interactions, potentially acting as structural ‘reinforcers’ that fill the gaps in the loose protein network induced by low ionic strength. Nevertheless, a persistent technical barrier remains as conventional marination often yields inefficient mass transfer, and these larger-molecule flavor-active components in SME may exhibit limited penetration into the dense and heterogeneous meat matrix.

Traditional static pickling primarily relies on concentration gradients to drive the diffusion of NaCl and other solutes through muscle tissue. This process is time-consuming, frequently produces variability in curing uniformity, and may be insufficient for the efficient delivery of bulky bioactive or flavor compounds [19]. To modernize this labor-intensive step, power ultrasound has emerged as a disruptive and practical technology. By generating acoustic cavitation, ultrasound induces cyclic compression/expansion and microstreaming, effectively “massaging” muscle fibers and accelerating solute diffusion [20]. Such effects have been reported to enhance salt diffusion in other curing systems, including pork [21]. Importantly, ultrasound can create or enlarge structural pathways within tissue that facilitate penetration of larger compounds. Despite these advantages, it remains unknown whether the micro-mechanical forces of US can facilitate the deep penetration of SME-derived functional components into the muscle fibers to effectively “bridge” the weakened protein network under reduced-sodium conditions. Therefore, the potential synergy between ultrasound-assisted marination and SME for improving the quality of reduced-sodium chicken breast has not been systematically clarified.

Therefore, the present study aimed to engineer a reduced-sodium marination strategy by combining shiitake mushroom extract with ultrasound-assisted pickling. In addition to macroscopic curing indicators (marinade uptake and chloride content), hematoxylin–eosin (H&E) staining and low-field nuclear magnetic resonance (LF-NMR) were applied to evaluate microstructural changes and water mobility within the muscle. Protein conformational responses were further assessed through surface hydrophobicity and sulfhydryl measurements to provide mechanistic insights. In short, this work seeks to establish a robust technological framework for developing sodium-reduced poultry products with improved curing efficiency without compromising quality.

## 2. Materials and Methods

### 2.1. Preparation of Shiitake Mushroom Extract

Fresh shiitake mushrooms were purchased from Jiajia Yue (Jinan, China). Food-grade sodium chloride was obtained from China National Salt Industry Group. Food-grade magnesium chloride and food-grade potassium chloride were supplied by Macklin Biochemical Co., Ltd. (Shanghai, China). All other analytical reagents were purchased from Sinopharm Chemical Reagent Co., Ltd. (Shanghai, China). Mushrooms were disinfected in a 200 ppm sodium hypochlorite solution for 10 min and rinsed with running water. Samples were cut into strips (1 cm width), steam-blanched for 3 min [22], and dried in a forced-air oven at 50 ± 2 °C to constant weight. Dried mushrooms were ground, passed through a 60-mesh sieve, and stored in a sealed light-protected container.

For extraction, mushroom powder was mixed with deionized water (1:20, *w*/*v*) and adjusted to pH 7.5. Ultrasound-assisted hot water extraction was performed using an ultrasonic bath (KQ5200E, Kunshan Ultrasonic Instrument Co., Ltd., Kunshan, China) at 40 kHz and 300 W in pulse mode (2 s on/1 s off), repeated twice. Extracts were centrifuged (SCR20BC, Hitachi, Tokyo, Japan) at 10,000× *g* for 15 min at 4 °C, and the supernatant was filtered through a 0.45 μm membrane. The filtrate was concentrated by vacuum evaporation at 40 °C (RE-52AA, Shanghai Yarong Bio-Chemical Instrument Factory, Shanghai, China) to one-fifth of the original volume and stored in a dry, light-protected environment until use.

### 2.2. Marination and Ultrasound Treatment

White-feathered chickens (reared for 60 days) were procured from Shandong Chunxue Food Co., Ltd., Laiyang, China. All visible connective tissue, adipose tissue, and skin were removed. Whole chicken breasts were cut into cubes measuring 3 cm × 3 cm × 2 cm, bagged, and stored frozen at −18 °C. All chicken breasts underwent a 24 h thawing process at 4 °C before testing. Samples were placed in polypropylene containers and marinated in six brine formulations: (i) 2% NaCl; (ii) 1.4% NaCl + 0.6% KCl; (iii) 1.4% NaCl + 0.2% KCl; (iv) 1.4% NaCl + 0.2% KCl + 0.4% mushroom extract; (v) 1.4% NaCl + 0.2% KCl + 0.8% mushroom extract; and (vi) 1.4% NaCl + 0.2% KCl + 1.2% mushroom extract. The meat-to-brine ratio was maintained at 150:250 (*w*/*v*).

Samples were assigned to either a non-ultrasound-treated group (T0–T5) or an ultrasound-treated group (UT0–UT5). For non-ultrasound treatments, samples were marinated at 4 °C for 90 min. The brine compositions for treatments T0–T5 corresponded to the six formulations listed above.

The ultrasonic and pickling methods have been slightly modified according to this approach [23,24]. For ultrasound treatments, brined samples were sonicated using a probe ultrasonicator (SCIENTZ-IID, Ningbo Scientz Ultrasonic Technology Co., Ltd., Ningbo, China) equipped with a 6 mm probe (20 kHz, 300 W). The probe was immersed in the brine and positioned 20 mm from the surface of the chicken breast samples. Sonication was conducted for 15 min with temperature control using an ice-water bath. Samples were manually rotated with intermittent pauses to ensure uniform ultrasonic exposure. Ultrasound-treated samples (UT0–UT5) corresponded to the same brine formulations as their respective non-ultrasound-treated counterparts. Following ultrasound treatment, all samples were sealed and further marinated at 4 °C for 75 min. Brine was drained prior to analysis. The control (T0) was marinated in 2% NaCl without ultrasound.

#### Rationale for Formulation Design

The salt formulations were designed to represent different strategies for sodium reduction. The 1.4% NaCl + 0.6% KCl group served as an ionic strength control, matching the total salinity of the 2.0% NaCl control. The 1.4% NaCl + 0.2% KCl formulation was established as a reduced-ionic-strength model, which is known to cause significant quality deterioration in poultry products (e.g., increased cooking loss and toughening). All SME-incorporated treatments were based on this 0.2% KCl formulation to specifically evaluate the potential of SME to act as a functional and flavor-compensating agent in a ‘worst-case’ reduced-sodium environment, thereby avoiding the bitterness associated with higher KCl concentrations [25].

### 2.3. Brine Uptake (Marinade Absorption)

To ensure accurate measurements, the surface moisture of the chicken samples was carefully removed by blotting with filter paper. Subsequently, the samples were weighed using an electronic balance to calculate the marinade absorption and cooking loss. Then, calculate the amount of marinade absorbed using the following Formula (1). m_1_ represents the initial mass, while m_2_ represents the weight after the seasoning has been absorbed.

(1) $$Marinade\;uptake\left(\%\right)=\frac{m_{1}-m_{2}}{m_{1}}\times 100$$

### 2.4. Chloride Content

Chloride content was determined using a modified titrimetric method based on Huang et al. [26]. A minced sample (10 g) was mixed with 50 mL deionized water (70 °C) in a 100 mL tube, heated in a 100 °C water bath for 15 min, ultrasonicated for 20 min, and cooled to 25 °C. Clarifying reagents were added sequentially: 2 mL Solution I (potassium ferrocyanide, 106 g/L) and 2 mL Solution II (zinc acetate 220 g/L + glacial acetic acid 30 mL/L). The mixture was brought to volume, mixed, and held for 30 min at 25 °C, then filtered. The filtrate was titrated for chloride using standardized AgNO_3_ solution (0.1 M) with potassium chromate indicator until a persistent brick-red endpoint. Chloride content was calculated as Formula (2).

(2) $$Chloride\;conted\left(\%\right)=\frac{0.0355\times C\times \left(V_{2}-V_{1}\right)\times 100}{m\times 50}\times 100$$

In this formula, C represents the concentration of the AgNO_3_ solution (mol/L), V_2_ represent the volume of the AgNO_3_ standard solution (mL), V_1_ represents the volume of the standard solution in the AgNO_3_ blank test (mL), 0.0355 represents the conversion factor of the AgNO_3_ standard solution (g) and m represents the weight of the sample (g).

### 2.5. Cooking Loss

Cooking loss was measured with minor modification of Li et al. [27]. The marinated chicken breast samples were vacuum-packaged and subjected to heat treatment in a programmed water bath (HHS-2, Sunshine Experimental Instruments Co., Ltd., Shanghai, China) maintained at 61 °C. A digital K-type thermocouple was inserted into the geometric center of the samples to monitor the thermal transition. The cooking process was terminated once the internal core temperature reached 60 °C. Following heat treatment, the samples were immediately transferred to an ice-water bath and cooled to a core temperature of 25 °C before further analysis. Cooking loss was calculated as follows (Formula (3)).

(3) $$Cooking\;loss\left(\%\right)=\frac{Raw\;weight-Cooking\;weight}{Raw\;weight}\times 100$$

### 2.6. Shear Force

Shear force was measured using the method described by Silva et al. [28] with a texture analyzer (EZ–TEST, Shimadzu, Kyoto, Japan). Each piece of meat was cut parallel to the muscle fiber direction (2 cm × 1 cm × 1 cm) for testing. The shear force was applied at right angles to the muscle fiber orientation. The maximal force registered was the shear force applied during the cutting of the chicken specimen. At least six points were tested in each piece of meat.

### 2.7. Myofibrillar Fragmentation Index (MFI)

MFI was determined following Delgado et al. [29]. Minced meat (5.0 g) was homogenized in 40 mL MFI buffer (0.1 M KCl, 10 mM K_2_HPO_4_, 2 mM MgCl_2_, 1 mM EGTA, 1 mM NaN_3_; pH 7.0) at 4 °C (three cycles of 30 s with 10 s intervals) using a homogenizer (FLUKO FA-25, Shanghai, China). The homogenate was filtered through 120-mesh gauze, and the suspension was adjusted to 0.5 mg/mL protein. The concentration was measured three times by spectrophotometry (UV756, Shanghai Youke Instruments Co., Ltd., Shanghai, China) at 540 nm. The mean result was then multiplied by 200 to calculate the MFI.

### 2.8. Hematoxylin-Eosin (HE) Staining

The muscle tissue fixed with paraformaldehyde becomes dehydrated and transparent. Three muscle tissues were randomly selected from each group for paraffin embedding. Fix the embedded paraffin blocks on a paraffin sectioning machine (LEiCA-RM2016, Shanghai Xuka Instrument Co., Ltd., Shanghai, China) with a thickness of 4 µm for sectioning. The sections were dewaxed and hydrated for hematoxylin-eosin (H&E) staining, dehydrated to become transparent, and then sealed. The sections were observed, and images were captured using an optical microscope (NIKON Eclipse Ci, Tokyo, Japan).

### 2.9. Low-Field Nuclear Magnetic Resonance Analysis (Low-Field NMR)

Low-field nuclear magnetic resonance relaxation measurements were performed using an LF-NMR analyzer (MesoMR23-060H, Niumag Analytical Instruments Co., Ltd., Suzhou, China) operating at 23 MHz. Measurements followed a previously described procedure [30].

### 2.10. Determination of Surface Hydrophobicity

Surface hydrophobicity was measured following the method described by Chelh et al. [31] with minor modifications. Meat paste (0.6 g) was homogenized in 20 mL phosphate buffer (20 mM, pH 6.0). BPB solution (200 μL, 1 mg/mL) was added to 1 mL homogenate, mixed, and incubated for 10 min. Samples were centrifuged at 6000 rpm for 15 min at 4 °C, and supernatant absorbance was measured at 595 nm against buffer blank. BPB bound was calculated as Formula (4):

BPB bound (μg) = 200 × (A reference − A sample)/A reference(4)

### 2.11. Determination of Reactive (Free) and Total Thiol Content

Reactive (free) and total thiol contents were determined using Ellman’s reagent (DTNB; 5,5′-dithiobis-(2-nitrobenzoic acid)) with minor modification [32]. For total thiols, 15 mg sample was suspended in 5 mL Tris–glycine buffer (pH 8.0) containing 8 M urea. For reactive thiols, 15 mg sample was suspended in 5 mL Tris–glycine buffer (pH 8.0) with urea. DTNB solution (50 μL, 10 mM) was added, mixtures were centrifuged at 5000× *g* for 15 min at 4 °C, and the supernatant was incubated at 25 °C for 1 h. Absorbance was measured at 412 nm. Thiol content was calculated as Formula (5).

SH (nmol/mg) = 73.53 × A × (D/C)(5)

where A is absorbance at 412 nm, D is dilution factor, and C = 13,600 M^−1^·cm^−1^.

### 2.12. Statistical Analysis

A total of 36 fresh chicken breasts (each from a different bird) were used for each of the 12 treatments. The experiment was organized into three independent batches (n = 3), utilizing fresh raw materials and newly prepared brine solutions for each batch. Within each batch, 12 chicken breasts were randomly assigned to account for biological variation. To avoid pseudo replication, the independent batch was defined as the experimental unit, while the individual chicken breasts served as observational sub-samples. All statistical analyses were performed using the mean values of these 12 sub-samples for each of the 3 independent batches. For quality attributes such as shear force, six individual measurements were taken per replicate, while for MFI and LF-NMR, measurements were performed in triplicate. The mean values from each independent replicate were used for the two-way ANOVA to determine the main effects and interactions. The normality of the distribution and the homogeneity of variance were verified using the Shapiro–Wilk test and Levene’s test, respectively. Data were analyzed using Two-way Analysis of Variance (ANOVA) via SPSS software (Version 26.0, IBM Corp., Armonk, NY, USA). The model included brine formulation and ultrasound treatment as fixed factors, as well as their interaction term. Significant differences between means were determined using Duncan’s multiple range test at a significance level of 0.05. Exact *p*-values and partial eta-squared ($\eta_{p}^{2}$) values were reported to indicate the level of significance and effect size, respectively.

## 3. Results and Discussion

### 3.1. Marinade Absorption and Chloride Content

**Figure 1:**
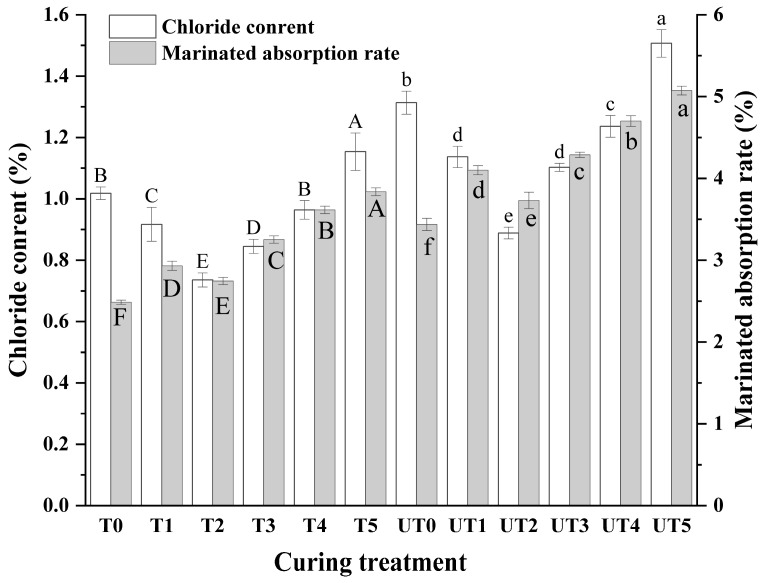
Effects of curing strategy on marinade uptake and chloride content of chicken breast. Treatments include non-ultrasound marination (T0–T5; 4 °C, 90 min) and ultrasound-assisted marination (UT0–UT5; 20 kHz, 300 W, 15 min) followed by standing at 4 °C for 75 min to maintain a constant total marination time (90 min). Brine formulations: T0/UT0, 2% NaCl (control); T1/UT1, 1.4% NaCl + 0.6% KCl; T2/UT2, 1.4% NaCl + 0.2% KCl; T3/UT3, T2 + 0.4% mushroom extract; T4/UT4, T2 + 0.8% mushroom extract; T5/UT5, T2 + 1.2% mushroom extract (*w*/*v*). Values are mean ± SD (n = 3). Uppercase letters denote significant differences (*p* < 0.05) among samples in the non-ultrasonic group; lowercase letters denote significant differences (*p* < 0.05) among samples in the ultrasonic group.

Figure 1 summarizes marinade absorption and chloride content of chicken breast under different curing strategies. Statistical analysis indicated that brine formulation was the primary driver for mass transfer (indicated by the higher $\eta_{p}^{2}$ value), though ultrasound provided a consistent synergistic boost. Compared with the 2% NaCl control (T0), partial substitution with KCl altered marinade absorption, with reduced-sodium treatments showing significantly higher uptake (*p* < 0.01). This response suggests that ionic composition influenced muscle hydration and the driving forces governing brine retention.

Addition of shiitake mushroom extract (0.4–1.2%) further modulated marinade absorption within the reduced-sodium system. Treatments containing mushroom extract generally showed higher uptake than the corresponding extract-free formulation at the same salt level (*p* < 0.01), which may be attributed to the presence of hydrophilic components and soluble solids that enhance water binding and swelling behavior in the meat–brine system [33].

Crucially, the significant interaction (*p_A × B_* = 0.002) highlighted that ultrasound-assisted treatments (UT0–UT5) produced formulation-dependent increases in mass transfer. Simple effect analysis showed that while ultrasound consistently improved absorption via acoustic cavitation and microstreaming [21], the most substantial gains were observed in the 0.8% SME group (UT4). In this specific combination, chloride content reached 1.23%, effectively narrowing the gap with the high-sodium control (T0). This statistically supports the 0.8% SME concentration as the optimal level for maximizing the synergistic benefits of power ultrasound in a low-ionic-strength environment.

### 3.2. Cooking Loss Analysis

Cooking loss differed significantly among treatments (Table 1 and Table 2), and two-way ANOVA confirmed significant main effects of brine formulation (*p* < 0.01, $\eta_{p}^{2}$ = 0.975) and ultrasound (*p* < 0.01, $\eta_{p}^{2}$ = 0.957), as well as their interaction (*p* < 0.01, $\eta_{p}^{2}$ = 0.832). In the reduced-sodium system, KCl substitution without mushroom extract produced higher cooking loss in specific formulations, indicating that salt reduction and altered ionic composition can weaken water immobilization during heating. By contrast, incorporation of mushroom extract significantly reduced cooking loss relative to extract-free reduced-sodium treatments at the same salt level, particularly at the higher extract levels where statistical differences were observed (*p* < 0.01). This finding suggests that mushroom extract improves water-holding capacity, potentially through enhanced protein–water interactions and additional hydrophilic binding sites contributed by extract components.

**Figure 2:**
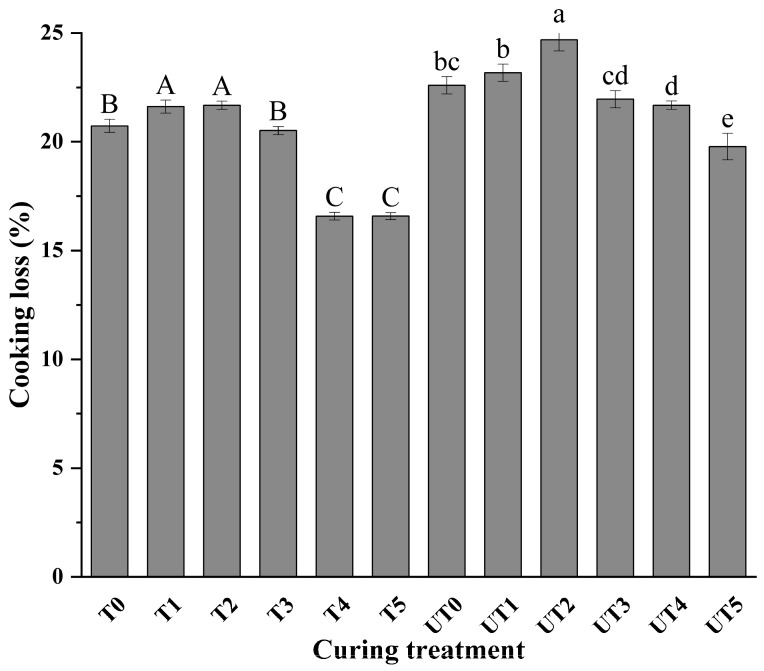
Effects of curing strategy on cooking loss of chicken breast. Treatment codes and brine formulations are as described in Figure 1. Values are mean ± SD (n = 3). Uppercase letters denote significant differences (*p* < 0.05) among samples in the non-ultrasonic group; lowercase letters denote significant differences (*p* < 0.05) among samples in the ultrasonic group.

The significant interaction effect (*p_A × B_* < 0.01) provides a statistical basis for the complex response of ultrasound assistance. Interestingly, ultrasound did not uniformly reduce cooking loss. While it enhanced brine uptake (Figure 2), in some low-SME formulations, ultrasound-treated samples showed comparable or slightly higher cooking loss than non-ultrasound counterparts. This suggests a trade-off: the intensive mechanical force of acoustic cavitation may cause localized structural disruption of muscle fibers; if the concentration of protective SME ‘reinforcers’ is insufficient, this disruption can lead to reduced thermal stability of the protein–water matrix [34].

However, simple effect analysis demonstrated that at the 0.8% SME level (UT4), the synergy between ultrasound and extract was maximized. In this treatment, the cooking loss was significantly lower than that of other reduced-sodium groups and showed no statistical difference compared to the 2.0% NaCl control (T0, *p* < 0.01). This indicates that 0.8% SME provides sufficient functional components to ‘bridge’ the ultrasound-induced pathways and stabilize the moisture within the meat matrix, statistically justifying this concentration as the optimal formulation for salt-reduced chicken breast.

### 3.3. Shear Force Analysis

**Figure 3:**
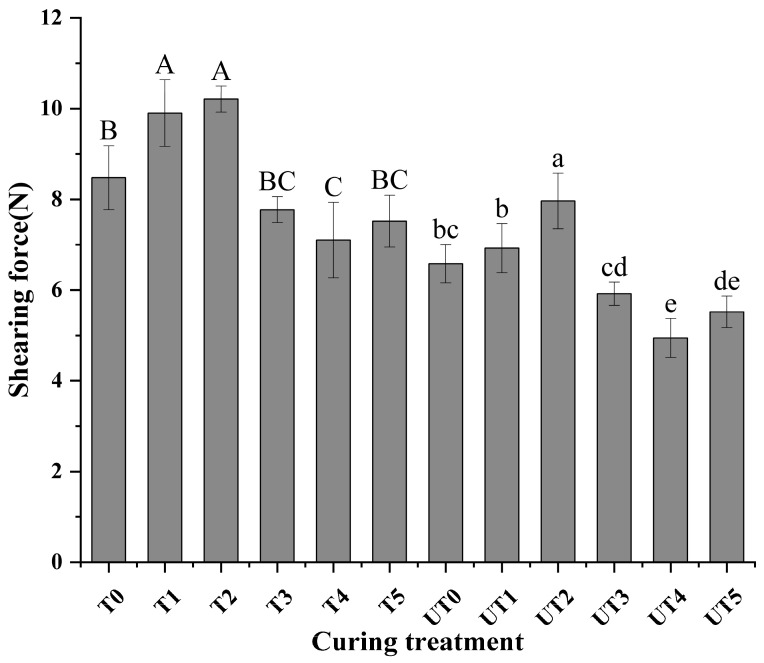
Effects of curing strategy on the shear force of chicken breast. Treatment codes and brine formulations are as described in Figure 1. Values are mean ± SD (n = 3). Uppercase letters denote significant differences (*p* < 0.05) among samples in the non-ultrasonic group; lowercase letters denote significant differences (*p* < 0.05) among samples in the ultrasonic group.

Shear force results are presented in Figure 3. Two-way ANOVA revealed (Table 1 and Table 2) that both brine formulation (*p*< 0.01, $\eta_{p}^{2}$ = 0.857) and ultrasound treatment (*p* < 0.01, $\eta_{p}^{2}$ = 0.863) exerted significant main effects on the shear force of chicken breast. However, no significant interaction was observed between these two factors (*p* = 0.495), suggesting that the tenderizing effects of shiitake mushroom extract (SME) and ultrasound were independent and additive. Addition of shiitake mushroom extract significantly reduced shear force in several reduced-sodium treatments compared with extract-free counterparts, indicating improved tenderness (*p* < 0.01). Similar tenderness improvements have been reported in mushroom-fortified meat products such as sausages and patties, which were attributed to altered hydration and matrix organization [17,35].

Ultrasound-assisted treatments further reduced shear force in multiple formulations (Figure 3), and significant differences between UT and T groups were observed where indicated (*p* < 0.01). This tenderizing effect is consistent with the mechanical disruption of the myofibrillar structure and weakening of fiber associations caused by acoustic cavitation and microstreaming. While these physical effects may facilitate the activity of endogenous proteases [36], our results primarily link the improved tenderness to structural fragmentation, as supported by the significant increases in the Myofibrillar Fragmentation Index (MFI) and the visible microstructural gaps.

Although the interaction was not statistically significant, the combined application of 0.8% SME and ultrasound (UT4) yielded the lowest numerical shear force among all reduced-sodium treatments. Notably, UT4 was the primary treatment that successfully reduced the shear force to a level comparable with the 2.0% NaCl control (T0). Therefore, from a functional perspective, the additive effects of 0.8% SME and ultrasound provide an effective strategy to compensate for the toughening typically associated with sodium reduction in poultry products.

### 3.4. Myofibrillar Fragmentation Index (MFI) Analysis

**Figure 4:**
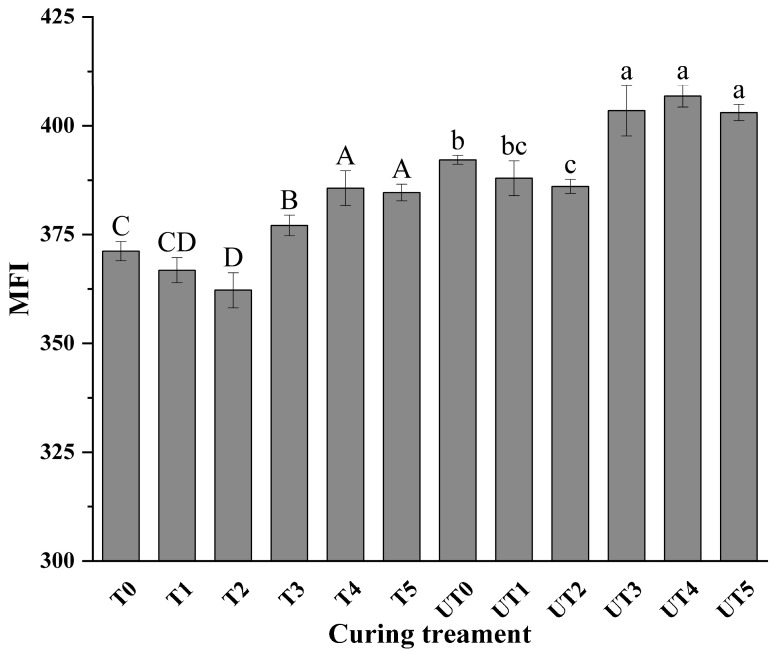
Effects of curing strategy on myofibrillar fragmentation index (MFI) of chicken breast. Treatment codes and brine formulations are as described in Figure 1. Values are mean ± SD (n = 3). Uppercase letters denote significant differences (*p* < 0.05) among samples in the non-ultrasonic group; lowercase letters denote significant differences (*p* < 0.05) among samples in the ultrasonic group.

MFI was evaluated as an indicator of myofibrillar disruption, where higher values generally reflect increased fragmentation near Z-discs and are often associated with improved tenderness [37]. Two-way ANOVA revealed (Table 1 and Table 2) that both brine formulation (*p* < 0.01, $\eta_{p}^{2}$ = 0.914) and ultrasound treatment (*p* < 0.01, $\eta_{p}^{2}$ = 0.949) exerted significant main effects on MFI. However, their interaction was not statistically significant (*p* = 0.346), suggesting that SME addition and ultrasound assistance promoted myofibrillar fragmentation through independent and additive pathways (Figure 4). In the reduced-sodium system, the addition of SME significantly altered MFI responses in a concentration-dependent manner. Compared with extract-free reduced-sodium brines, SME-containing treatments generally exhibited higher MFI values (*p* < 0.01). This suggests that SME components may facilitate the solubilization of myofibrillar proteins or weaken the structural integrity of the sarcomere, rendering it more susceptible to fragmentation during homogenization.

Notably, ultrasound-assisted treatments with mushroom extract displayed increased MFI compared with their corresponding non-ultrasound counterparts in multiple formulations (*p* < 0.01), indicating that ultrasound promoted fragmentation and structural weakening of myofibrils. These results are consistent with the physical disruption produced by cavitation and localized shear, which can facilitate myofibrillar disassembly and protein dissolution [38,39], thereby contributing to tenderness improvements observed in Figure 3. While the interaction was not significant, the combined application of 0.8% SME and ultrasound (UT4) resulted in the highest numerical MFI among the reduced-sodium groups. These findings are consistent with the shear force reductions (Figure 3) and microstructural observations, collectively supporting the conclusion that the additive effects of SME and ultrasound effectively enhance the fragmentation of the muscle matrix under sodium-reduced conditions.

### 3.5. Hematoxylin-Eosin (H&E) Staining Analysis

**Figure 5:**
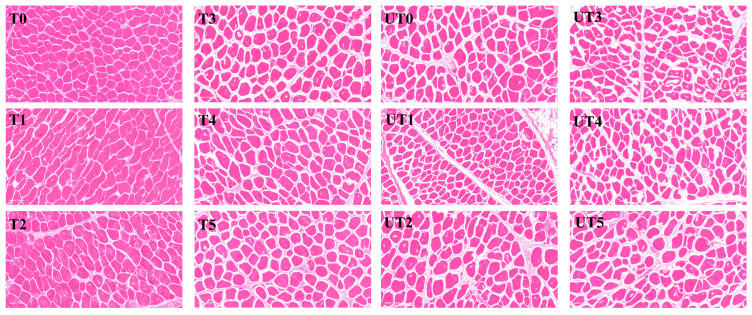
Representative H&E micrographs showing muscle fiber structure of chicken breast after marination with or without ultrasound assistance. Treatment codes and brine formulations are as described in Figure 1.

Microstructural changes revealed by H&E staining are shown in Figure 5. The control muscle (T0) exhibited tightly packed fibers with limited inter-fiber gaps, which is consistent with osmotic dehydration and protein contraction associated with high NaCl conditions. When NaCl was partially replaced by KCl (T1–T2), the organization of muscle bundles became less compact, and inter-fiber spaces appeared enlarged. This response is plausibly related to differences in ionic interactions and hydration behaviors of K^+^ compared with Na^+^ within the myofibrillar protein matrix [40].

**Figure 6:**
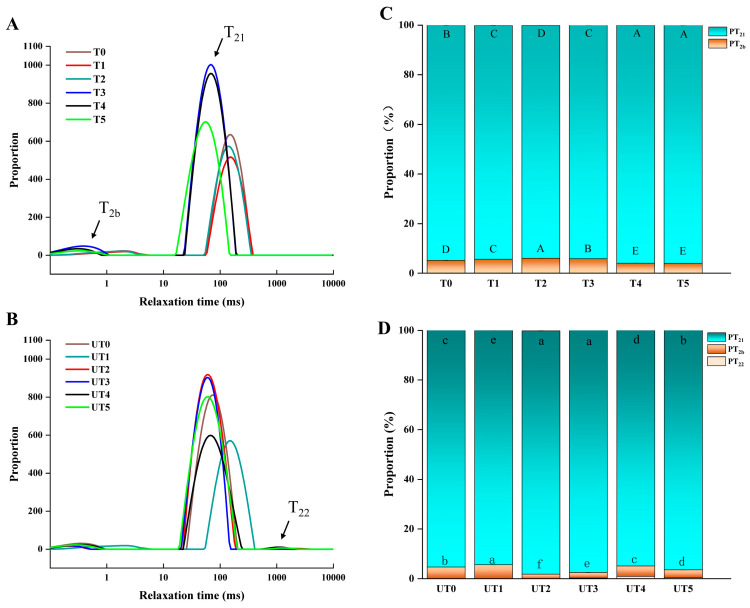
Low-field NMR analysis of water mobility and distribution in chicken breast subjected to different curing strategies: (**A**,**B**) T_2_ relaxation times; (**C**,**D**) peak area ratios corresponding to T_2_ components. Treatment codes and brine formulations are as described in Figure 1. Values are mean ± SD (n = 3). Uppercase letters denote significant differences (*p* < 0.05) among samples in the non-ultrasonic group; lowercase letters denote significant differences (*p* < 0.05) among samples in the ultrasonic group.

Addition of mushroom extract (T3–T5) further promoted a more open and relaxed structure, and the degree of structural loosening varied with extract level. The overall tendency toward increased spacing supports the improved water retention observed in Figure 2 and the altered water distribution shown by LF-NMR (Figure 6). Consistent with the significant formulation × ultrasound interaction (*p* < 0.01) observed in cooking loss, the micrographs suggest that an intermediate extract level (0.8%) produced a more uniform loosening pattern. Simple effect analysis confirmed that the 0.8% SME + ultrasound combination (UT4) resulted in significantly greater structural improvement than the 0.4% groups (*p* < 0.05), while providing no statistical difference compared to the 1.2% group. This statistical parity identifies 0.8% as the optimized balance point between hydration and matrix stability [41]. This visual evidence aligns with the statistical findings that 0.8% SME provides the most effective structural reinforcement in a low-sodium environment.

Ultrasound-treated samples showed the most pronounced structural modification (UT groups), characterized by enlarged gaps between fascicles and more evident disruption of fiber integrity relative to the corresponding non-ultrasound groups. Cavitation and shear forces can weaken connective boundaries and are reported to generate a “micro-pumping” effect that drives brine constituents deeper into the tissue [21]. These microstructural changes provide the physical basis for the significant main effects of ultrasound observed in marinade absorption and chloride content (Figure 1), as well as the independent additive effects found in shear force and MFI.

### 3.6. Moisture Distribution and Fluidity

LF-NMR was used to characterize water mobility and distribution in the marinated chicken breast, and the results are shown in Figure 6. The transverse relaxation time (*T*_2_) profiles typically reflect three water populations: *T*_2*b*_ (bound water tightly associated with macromolecules), *T*_21_ (immobilized water constrained within myofibrillar structures), and *T*_22_ (more mobile/free water) [42,43]. Two-way ANOVA indicated (Table 1 and Table 2) significant main effects of brine formulation (*p* < 0.01) and ultrasound (*p* < 0.01) on *T*_21_ relaxation times and their corresponding peak area ratios (*P*_21_), with a significant interaction observed for *P*_21_ (*p_A_*_× *B*_ < 0.01).

A clear difference in relaxation behavior was observed between non-ultrasound (T) and ultrasound (UT) groups (Figure 6A,B). In several formulations, ultrasound shifted *T*_2_ parameters in a manner consistent with increased water mobility and structural opening. Longer relaxation times can reflect reduced constraints on water protons and may be associated with an expanded tissue network and increased inter-fiber space [44]. These results agree with the microstructural observations (Figure 5), indicating that ultrasound enlarged physical pathways and altered the local environment controlling water migration and entrapment [45]. Such optimized water mobility is a critical indicator of structural stabilization, as recently modeled by Bernardo et al. [46], who demonstrated that high-intensity ultrasound effectively modifies the porosity of muscle tissue, allowing for better retention of functional ingredients within the interstitial spaces.

In addition, mushroom extract addition significantly modified *T*_21_ and *T*_2*b*_, their corresponding peak area ratio in several reduced-sodium treatments (Figure 6C,D; *p* < 0.01). The observed interaction effect (*p_A × B_* < 0.01) highlights a synergistic mechanism: while ultrasound facilitates the penetration of brine, the SME components (e.g., polysaccharides) effectively ‘trap’ this moisture within the myofibrillar network. Specifically, the shortening of *T*_21_ and the increase in *P*_21_ proportion in extract-containing groups suggest strengthened interactions between water and the protein matrix. Simple effect analysis confirmed that the 0.8% SME + ultrasound combination (UT4) achieved the highest *P*_21_ value, indicating the most effective water immobilization among all reduced-sodium groups, which directly supports the minimum cooking loss observed in Figure 2. In summary, LF-NMR results provide statistical and physical evidence that SME improves water immobilization, whereas ultrasound regulates water redistribution, with their combination at 0.8% SME providing an optimal balance for moisture retention in low-sodium chicken breast.

### 3.7. Surface Hydrophobicity

**Figure 7:**
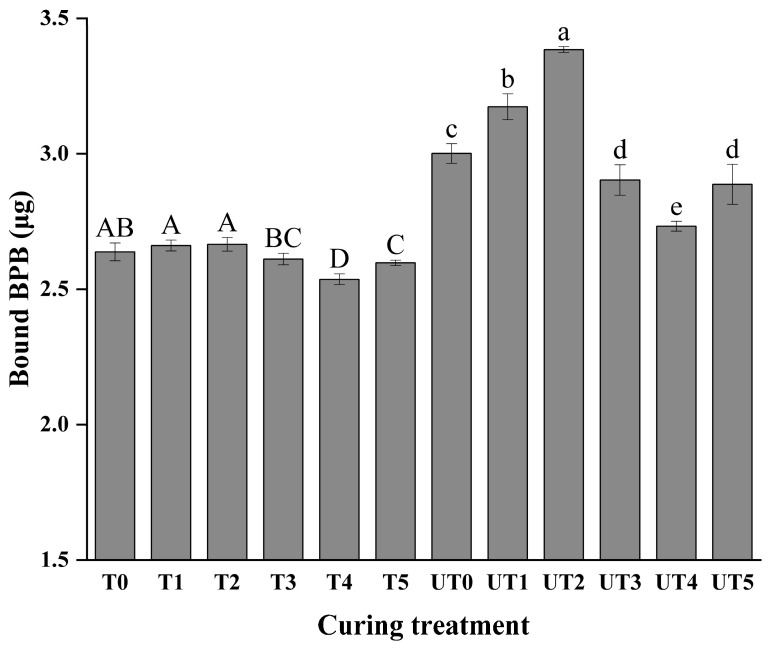
Surface hydrophobicity of chicken breast proteins following different marination treatments. Treatment codes and brine formulations are as described in Figure 1. Values are mean ± SD (n = 3). Uppercase letters denote significant differences (*p* < 0.05) among samples in the non-ultrasonic group; lowercase letters denote significant differences (*p* < 0.05) among samples in the ultrasonic group.

Protein surface hydrophobicity is closely related to functional properties of meat proteins, including hydration, emulsification, and gel formation, and it can strongly influence water retention in cooked products [47]. As shown in Figure 7, two-way ANOVA revealed significant main effects for both brine formulation (*p* < 0.01) and ultrasound treatment (*p* < 0.01), as well as a significant interaction between these two factors (*p* < 0.01). KCl substitution alone did not produce a significant change in surface hydrophobicity compared with the control in certain cases, whereas the addition of mushroom extract caused a significant decrease in hydrophobicity in several formulations (*p* < 0.01). A reduction in hydrophobicity suggests fewer exposed hydrophobic residues and/or increased exposure of polar groups, which can enhance protein–water interactions and is consistent with the improved cooking stability reflected by lower cooking loss (Figure 2).

In contrast, ultrasound treatment increased surface hydrophobicity in multiple formulations (Figure 7; *p* < 0.01). This effect is commonly attributed to ultrasound-induced partial unfolding of myofibrillar proteins, which exposes internal hydrophobic side chains (e.g., Leu, Ile, Tyr) and increases measurable hydrophobicity [48,49]. This result is similar to the findings reported by Robaló et al. [50]. The significant interaction (*p_A × B_* < 0.01) indicates that SME components may have a “shielding” or stabilizing effect on the unfolded protein structure, modulating the extent of hydrophobic group exposure. This protein-level response provides a mechanistic explanation for the observation that ultrasound can improve curing efficiency (Figure 1) and tenderness (Figure 3) while not necessarily maximizing water retention during heating (Figure 2), because excessive unfolding and structural disruption may reduce the stability of the water-holding network under thermal conditions. Simple effect analysis further highlighted that at 0.8% SME (UT4), the balance between ultrasound-induced unfolding and SME-mediated stabilization reached an optimal state, supporting the superior functional properties observed at this concentration.

### 3.8. Reactivity (Free) and Total Thiol Content

**Figure 8:**
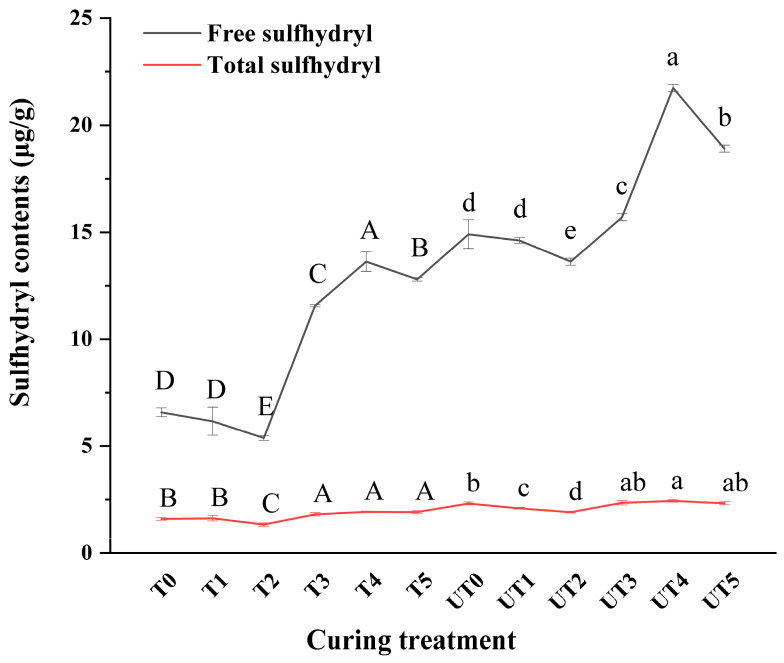
Reactive (free) and total sulfhydryl contents of chicken breast proteins after different curing strategies. Treatment codes and brine formulations are as described in Figure 1. Values are mean ± SD (n = 3). Uppercase letters denote significant differences (*p* < 0.05) among samples in the non-ultrasonic group; lowercase letters denote significant differences (*p* < 0.05) among samples in the ultrasonic group.

Sulfhydryl (thiol) groups exist either as reactive surface-exposed residues or as buried residues that become detectable after denaturation, and thiol status is therefore informative for protein conformational changes and oxidative cross-linking [36]. As shown in Figure 8, both reactive (free) and total sulfhydryl contents differed among treatments. Mushroom extract supplementation produced a non-linear response, with two-way ANOVA revealing that both brine formulation and ultrasound significantly influenced reactive thiol content (*p* < 0.01). Specifically, simple effect analysis confirmed that reactive thiol content was highest at the intermediate extract level (0.8%) in the reduced-sodium system (*p* < 0.01). This statistical optimum suggests that this condition may favor thiol exposure and/or preservation. Such a pattern is hypothesized to reflect a balance between protein unfolding (increasing accessibility of thiols) and potential structural modifications as extract concentration changes. While these shifts align with the significant interaction effect (*p_A × B_* < 0.01) observed in surface hydrophobicity (Figure 7), further molecular analysis would be required to definitively differentiate between the effects of protein unfolding and specific oxidative or cross-linking pathways.

Ultrasound further modified sulfhydryl profiles. In several cases, total sulfhydryl content increased after ultrasound treatment (*p* < 0.01), which may reflect increased accessibility of previously buried thiol groups under denaturing measurement conditions due to ultrasound-driven conformational expansion. At the same time, ultrasound-generated cavitation can produce localized radicals that promote thiol oxidation and disulfide bond formation [51]. Therefore, the combined thiol results, together with surface hydrophobicity (Figure 7) and LF-NMR outcomes (Figure 6), suggest a potential mechanistic model in which ultrasound initially promotes unfolding and permeability. It is hypothesized that an appropriate mushroom extract level (notably 0.8%) assists in stabilizing protein networking and water immobilization, as evidenced by the significant interaction effects observed in hydrophobicity and *P*_21_ distribution (*p* < 0.01). While this stabilization may involve enhanced protein–protein interactions or the formation of disulfide cross-links [52,53], further molecular studies are required to directly confirm these specific chemical modifications.

## 4. Conclusions

Driven by the global public health initiative to reduce dietary sodium, the poultry industry faces the critical challenge of balancing human health benefits with the maintenance of high food quality. To address this ‘quality-health trade-off’, this study demonstrates that combining shiitake (*Lentinula edodes*) mushroom extract with ultrasound-assisted marination provides an effective, clean-label approach to enhance curing efficiency and quality attributes in reduced-sodium chicken breast. Two-way ANOVA revealed significant interactions (*p* < 0.05) between brine formulation and ultrasound, particularly for water retention and protein conformation. The mushroom extract improved marinade absorption and enhanced water-holding capacity, while ultrasound intensified mass transfer and increased chloride uptake, consistent with a more porous and disrupted muscle microstructure. LF-NMR and H&E staining confirmed that 0.8% extract with ultrasound optimized water immobilization and tissue openness. Protein-level responses suggest that ultrasound-induced unfolding was balanced by extract-mediated stabilization; however, specific chemical mechanisms like disulfide-mediated networking are identified here as hypotheses requiring further study. Despite the significant functional improvements observed, this study has certain limitations. First, while the macroscopic and microstructural changes were well-characterized, the precise molecular binding sites and dynamic interactions between the mushroom extract components and myofibrillar proteins under ultrasonic fields require further elucidation using more advanced molecular docking or specific spectroscopic tools. Second, the current findings are based on controlled, laboratory-scale batch marination. The thermodynamic behavior and mass transfer efficiency of this synergistic system under continuous industrial-scale processing remain to be validated. Future research addressing these specific aspects will further facilitate the successful industrial transition of this sodium-reduction strategy.

## Figures and Tables

**Table 1:** *p*-values of the interaction effect between ultrasonic treatment and salt water ratio on chicken breast meat indicators.

	Characterization	Brine Uptake (Marinade Absorption)	Cooking Loss	Shear Force	MFI	Low-Field NMR	Surface Hydrophobicity	Mercapto Group
*p*								
Ultrasound treatment (A)		*p* < 0.01	*p* < 0.01	*p* < 0.01	*p* < 0.01	*p* < 0.01	*p* < 0.01	*p* < 0.01
Saline ratio (B)		*p* < 0.01	*p* < 0.01	*p* < 0.01	*p* < 0.01	*p* < 0.01	*p* < 0.01	*p* < 0.01
Interaction (A × B)		*p* = 0.002	*p* < 0.01	*p* = 0.495	*p* = 0.346	*p* < 0.01	*p* < 0.01	*p* < 0.01

**Table 2:** The partial eta-squared ($\eta_{p}^{2}$ values for the main effects (ultrasound treatment and saline ratio) and their interaction effect.

	Characterization	Brine Uptake (Marinade Absorption)	Cooking Loss	Shear Force	MFI	Low-Field NMR	Surface Hydrophobicity	Mercapto Group
$\eta_{p}^{2}$								
Ultrasound treatment (A)		0.951	0.957	0.863	0.948	0.985	0.978	0.995
Saline ratio (B)		0.968	0.975	0.857	0.914	0.982	0.948	0.992
Interaction (A × B)		0.526	0.832	0.158	0.198	0.99	0.897	0.9

## Data Availability

The data presented in this study are available on request from the corresponding author due to privacy concerns.
